# Complexity of biological scaling suggests an absence of systematic trade-offs between sensory modalities in *Drosophila*

**DOI:** 10.1038/s41467-022-30579-y

**Published:** 2022-05-26

**Authors:** Max S. Farnworth, Stephen H. Montgomery

**Affiliations:** School of Biological Sciences, University of Bristol, Bristol, UK

**Keywords:** Evolutionary developmental biology, Development of the nervous system, Sensory processing

**arising from** Ian W. Keesey et al. *Nature Communications* 10.1038/s41467-019-09087-z (2019)

The structure of nervous and sensory systems reflects the interactions between selection pressures and functional integration, developmental constraints, and energetic costs^1,2^. How these interactions play out over time is a central question in evolutionary neurobiology, put simply; how do brains evolve, and why do they evolve that way? To address this question, a range of data is needed, spanning development, detailed anatomy and behaviour, ideally across ecologically diverse clades of species to examine how traits change at a phylogenetic scale^3^. Recently, Keesey et al.^4^ provided a taste of the kind of expansive, integrative studies required. By combining data on sensory structures across ~60 *Drosophila* with neuroanatomical, behavioural and developmental data from selected species, they provide an in-depth examination of the evolution of *Drosophila* sensory and brain structure. A central conclusion drawn from these analyses was that visual and olfactory structures appear to be consistently targeted for expansion at the expense of one another – both in peripheral and central neural tissue. This hints at a pervasive underlying constraint on the sensory biology of *Drosophila* imposed by developmental trade-offs in the eye-antennal imaginal disc. Such a trade-off could suggest that some trait combinations are unobtainable, potentially impacting broader ecological patterns across the genus^5^. Here, we suggest the approach taken in the analyses negates key features and major shifts in biological scaling^6^, which, when re-examined in detail, present a more complex pattern of neural diversity, inconsistent with systematic trade-offs.

One central issue of the original paper is that the conclusions were drawn in part from an examination of trait ratios, for example the ratio of eye size over a measure of antennal size. Ratios can often be problematic as they assume an isometric scaling relationship between the numerator and denominator, because they set one trait in proportion to the other (i.e. ^y^/_x_)^6^. Isometric scaling between two traits reflects a slope (*β*) of 1 in a classical allometric relationship *log(y) = βlog(x) + α*. Where the true scaling relationship deviates from 1, as may often be the case in biological systems^7^, this introduces biases in how the relative size of a trait is measured, with hyper-allometry of the numerator (*β* > 1) causing inflation of the ratio with increasing organismal size, while hypo-allometry (*β* < 1) would have the opposite effect^6^ (see also Supplementary Note 1). This limitation can be overcome by including an allometric control in a multiple regression alongside the traits of interest (e.g., vision ~ body size + olfaction), as is common practice in allometric studies^8^. Here, we use such an approach to re-evaluate evidence for systematic trade-offs between sensory modalities during the diversification of *Drosophila*. In particular, we focus on the following predictions that are made under the assumption of a developmental trade-off model: i) the two traits should not vary independently across time; ii) trait scaling relationships should be conserved; iii) negative trait correlations should be present within as well as between species if conserved developmental ties are constraining interspecific variation, and iv) these relationships should be reflected in both developing and adult tissue.

Our starting point was the most phylogenetically broad dataset, which quantified variation in eye surface area (ESA) and the third antennal segment, the funiculus (FSA). Using data from 59 *Drosophila* species, Keesey et al.^4^ calculated the ratio of these traits (the EF ratio), to characterise species as visually orientated (high EF ratio) or olfactorily orientated (low EF ratio). Although the EF ratio is reported to show associations with some behavioural or morphological traits, several assumptions are made by this approach: (i) that the ratio accurately captures variation along a common scaling relationship; (ii) that subsequent associations with other behavioural and developmental traits are explained by the size of ESA and FSA relative to one another; and (iii) that ESA and FSA cannot vary independently (Fig. 1A). Focusing on the latter, we reanalysed the data using phylogenetically controlled regressions that appropriately estimate scaling relationships^9,10^. We found that, even when accounting for body size or head size (Supplementary Note 3), ESA and FSA scale allometrically (*β* = 0.759, *t*_df=54_ = 6.124, *P* < 0.001, Fig. S1). Sub-setting the data to smaller monophyletic groups leads to the same conclusions (Supplementary Note 2). This result suggests these structures vary in size consistently, but in a positive direction. Consistent with an absence of a negative association, when each is regressed against body size to remove allometric effects (Fig. 1B), the residual variances in ESA and FSA are also positively associated (*β* = 0.811, *t*_df=57_ = 6.937, *P* < 0.001; Fig. 1C). This contradicts prior claims of inverse relationships under the scenario of a trade-off, which may be explained by the obscuring effect of ratios, which can mask independent patterns of variation (Fig. 1A). We also note that because ESA is a much larger number than FSA it has a dominant effect on the EF ratio. Indeed, ESA and EF covary positively with each other (*t*_df=56_ = 3.237, *P* = 0.002), suggesting the EF ratio fails to remove all allometric effects, a predicted limitation of using ratios as a measure of relative size^6,11^. We therefore suspect that the behavioural associations found for the EF ratio may largely be explained by variation in the absolute size of ESA, and not by the balance, or ratio, between ESA and FSA.

**Fig. 1:**
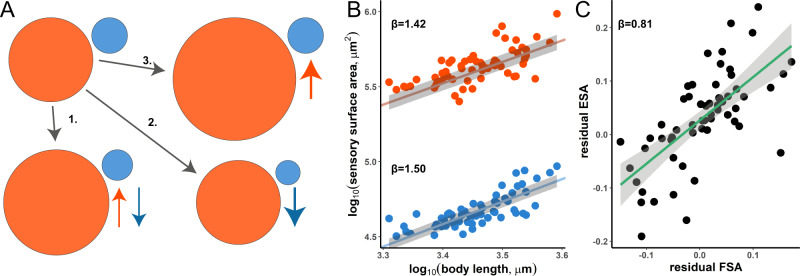
Analysing scaling relationships of eye and funiculus surface area [ESA (red) and FSA (blue)] reveals lack of inverse relationship between vision and olfaction in 59 species. **A** Using ratios does not distinguish between a trade-off scenario (1.) from others where either vision (numerator) is strongly increased (2.) or olfaction (denominator) strongly decreased (3). Each scenario presented can result in the same EF ratio of 11.81 (the highest value in the dataset) when converted from a ratio of 5.54 (the lowest value in the dataset), where the circle area reflects the volume used to determine the EF ratio. **B** MCMC based phylogenetic regression models in BayesTraits V3.0.2^10^ of both surface areas reveals allometric scaling with body length. Indicated is the slope of each regression line based on phylogenetically controlled regressions, with confidence intervals as grey bands. **C** Calculating the residuals from B, performing a phylogenetic linear regression and plotting them reveals a positive relationship, i.e. an absence of an inverse relationship (confidence intervals as grey bands). Source data are provided as a Source Data file.

Similar inverse allocation trade-offs were also reported among brain components that process visual and olfactory information, in the optic lobe (OL) and antennal lobe (AL) respectively. Here, although the data is phylogenetically less broad, specifically focusing on species with a range of EF ratios, it has the advantage of including more individuals per species, which permits analysis of both intra- and inter-specific scaling. We first analysed all individual data together, with species as a fixed effect in a generalized linear model (GLM) to determine if OL and AL show consistent scaling relationships across species, and/or evidence of negative relationships. Although both OL and AL scale allometrically (with the slope *β* of ~0.75) with the volume of the rest of the brain (RoB) in independent models (OL~RoB: *t*_df=22_ = 4.107, *P* < 0.001; AL~RoB: *t*_df=22_ = 3.083, *P* = 0.005; Fig. S2A/A′), when included in a model controlling for RoB we find no significant association between OL and AL (*t*_df=21_ = 0.575, *P* = 0.571). To visualise this result, we calculated residual variance around independent AL~RoB and OL~RoB regressions and plotted these values, which again shows no consistent relationship (*t*_df=27_ = 0.652, *P* = 0.520; Fig. S2A″). This suggests that although both visual and olfactory neuropil scale with overall brain size, once these general allometric effects are removed, the OL and AL vary independently of each other across. Indeed, in all models, species was a significant effect, also implying the intra-specific scaling relationships vary across species. Again, this is contrary to what would be predicted under a trade-off model; first, this model would expect the evolution of these structures to be closely correlated, albeit negatively, and second, to form a consistent constraint across evolutionary timescales the scaling relationships in between species would also be expected to be conserved.

To further investigate this variation in intraspecific scaling, we used SMATR^12^ to calculate interspecific differences in the slope and elevation of scaling relationships between OL, AL and RoB. In general, we found that, although the slopes of the scaling relationships were conserved, the intercepts varied significantly between species (OL: *W*_df=5_ = 513.100, *P* < 0.001; AL: *W*_df=5_ = 15.150, *P* = 0.010). If these shifts in OL and AL size were explained by developmental trade-offs we would expect that pairwise shifts for one structure would be inversely mirrored in the other. For example, if a species had a significantly higher elevation for OL~RoB over another species, it would show a significantly lower elevation for AL~RoB, as developing tissue is reallocated from one structure to the other. In pairwise comparisons between all six species, although there are some cases where this is observed (2 of 15; see off-diagonal of Fig. 2C, D for shift directions), we also observe shifts that occur specifically for one structure but not the other (Fig. 2A/C, B/D). For example, we detect a significant shift between *D. funebris* and *D. busckii* for OL~RoB, but not for AL~RoB, while between *D. pseudotalamancana* and *D. americana* we detect a shift in AL~RoB but not OL~RoB.

**Fig. 2:**
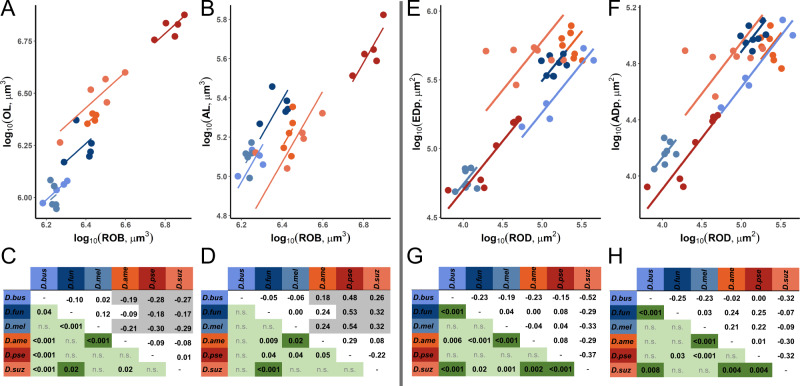
Analysing scaling relationships of vision and olfaction related structures in the brain and eye-antennal imaginal discs reveals a lack of inverse relationships between vision and olfaction despite strong variation in scaling between six *Drosophila* species. (**A**–**B**, **E**–**F**) The top row displays a plot of the visual and olfactory portion against the relevant allometric control (Rest of Brain, RoB, or Rest of Disc, RoD). Regression lines were derived from a SMATR^12^ analysis, where slope differences could not be detected. The analysis revealed intercept differences, after which a species pair-wise analysis was conducted. (**C**–**D**, **G**–**H**) This pair-wise analysis (based on the *Wald* statistic) is displayed in the bottom row. The green diagonal displays the significance of pair-wise differences. If the pair showed significant differences in the visual and olfactory portion this is indicated by dark green. The off diagonal indicates the difference between the intercepts. Opposing signs of intercept differences between **C** and **D** or **G** and **H** could indicate an inverse relationship between a specific species pair where one structure is traded off against another. Cases which fit this description and show a shift are indicated by a grey background (excluding pair-wise comparisons with minor (<0.1 units) differences). Abbreviations: OL: optic lobe, AL antennal lobe, ROB rest of brain, EDp eye disc portion, ADp antennal disc portion, ROD rest of disc, n.s. not significant; *D.bus* = *D. busckii* (*N* = 4 brains*; N* = 5 imaginal discs), *D.fun* = *D. funebris* (*N* = 5; *N* = 8)*, D.mel* = *D. melanogaster* (*N* = 5; *N* = 7)*, D.ame* = *D. americana* (*N* = 5; *N* = 7)*, D.pse* = *D. pseudotalamancana* (*N* = 5; *N* = 7)*, D.suz* = *D. suzukii* (*N* = 5; *N* = 7). Source data are provided as a Source Data file.

Similar analyses can also be performed on data on the relative size of the eye (EDp) and antennal (ADp) portions of the eye-antennal imaginal disc. Here, previous analyses suggested a potential trade-off between these developmental tissues, however, these data were again analysed as a ratio, which might obscure more complex patterns of size variation. To explore this possibility, we calculated a value for the overall size of the imaginal disc, minus the EDp and ADp, to give an independent size control (Rest of Disc, RoD, Fig. S3G). EDp and ADp scale positively with one another, even when RoD is included in the regression model (*t*_*df=33*_ = 18.213, *P* < 0.001, Fig. S3A–D’), and when also included as a fixed effect, species was a significant factor, again indicating interspecific variation in scaling relationships (ANOVA: *F* = 64.598, *P* < 0.001). Using SMATR we explored how intraspecific scaling relationships vary between species. As with the adult neuropil data, most variation stemmed from elevation shifts (EDp: *W*_df=5_ = 27.240, *P* < 0.001; ADp: *W*_df=5_ = 44.140, *P* < 0.001), and we find pairwise patterns of variation that are inconsistent with straightforward allocation trade-offs (Fig. 2E/G, F/H). Although there may be limitations to the extent that this data capture potential changes in the subdivision of the eye-antennal imaginal disc, this again implies that, across species, based on the available data, tissues contributing to structures involved in different sensory modalities are not consistently traded-off against one another. A dedicated comparison of disc development might be able to more conclusively answer questions about the scaling relationships in this developmental primordium.

Keesey et al.^4^ also supported their conclusions with evidence from genetic mutants that show at least one locus affecting the development of olfactory and visual traits in opposite directions, as suggested in an independent study^13^. We do not disagree that some loci could affect development in this way, but would note that evidence of potential developmental mechanisms does not necessarily reflect evolutionary trajectories. If heritable variation exists both in pathways that affect traits independently and in pathways that affect both in conjunction, selection could act on either, depending on the relative fitness costs/benefits^14^. Nevertheless, we reason that any persistent developmental or genetic trade-offs should manifest themselves within, as well as between, species. But with both the adult and developmental disc data, residual variance in OL and AL, or EDp and ADp, calculated from regressions with the relevant allometric control, show few instances of significant negative trait associations (Fig. S2C and S3F). Hence, these data further imply a degree of independent variation between sensory modalities within species that is not readily compatible with a model of developmental or evolutionary trade-offs.

In sum, our reanalyses suggest that the adoption of trait ratios masked complex patterns of biological scaling that are not consistent with the hypothesis that structures underlying different sensory modalities are consistently expanded at the expense of another, and are therefore unlikely to be traded-off due to shifting balances of a shared developmental mechanism. While such a trade-off mechanism in a common primordium might in some contexts have advantageous fitness effects through its mechanistic simplicity^14^, inferring fitness consequences of developmental change is fraught with difficulty and rarely tested. Instead we suggest the data are more consistent with a pattern of evolution in which scaling relationships are generally conserved, but with the scope for targeted expansions or reductions in brain components independently of one another, where selectively advantageous. Where coincident and diametric shifts in visual and olfactory investment do occur, based on the available evidence this could just as equally reflect negatively correlated selection pressures on sensory systems rather than direct trade-offs^14^.

However, our reanalysis also reveals a deficit of power to detect allometric shifts between species with the current sampling, with some shifts being visually apparent but not statistically supported due to low sample size (Figs. 2, S2C and S3F). We also note that if pairwise elevation differences are plotted against one another for the OL and AL scaling relationships (the y-intercepts in Fig. 2A, B), we do recover a negative relationship (Fig. S2B). This may suggest a more general tendency for shifts in these traits to occur in opposite directions. However, such explorative analysis has several caveats: i) this analysis involves comparing elevation differences that are not statistically significant; ii) as a result of the low sample size the confidence intervals around these estimates are also very large; iii) the pairwise correlations are not phylogenetically independent; and iv) we also recover a positive association for a similar analysis with EDp and ADp (Fig. S3E), which is inconsistent with the result for OL/AL. This uncertainty highlights that while extensive phylogenetic sampling is beneficial for generalisation across evolutionary time, large intra-specific datasets are also needed to fully explore these macro-evolutionary patterns.

Understanding how brains evolve is a daunting challenge in which a researcher must themselves reconcile trade-offs between time spent accruing data in one species, impacting the level of biological sampling and sample size, and time spent broadening the phylogenetic scope of the data, impacting the power to generalise across evolutionary time^15^. Keesey et al.’s^4^ pioneering study undoubtedly demonstrates the benefits of combining neuroanatomy, behaviour and development, and setting these in a clear, phylogenetic viewpoint. Building on this work, it will be critical for future studies to also consider maximising the scope for robust controls of allometric effects, enabling statistical models to probe independent contributions of different factors of neural variation to ecological and behavioural evolution, and as such avoiding to use ratios in evolutionary studies altogether^6^. This approach, combined with an ever-growing tool kit for high throughput behavioural and neural analyses, and the broadening scope for probing nervous systems with genetic tools^16^, sets an exciting trajectory for understanding the mechanisms and processes that underpin behavioural diversity in which comparative and experimental/functional studies can provide complementary tests of evolutionary hypothesis.

## Methods

We used MCMC based phylogenetic regression models in BayesTraits V3.0.2^11^ to analyse the head sensory structure dataset, and linear models available through R (version 4.1.1^17^) standard packages as well as species-specific slopes tests using the R package *smatr*^12^ for the brain and imaginal disc datasets (see Supplementary Code 1 file). For more detail, please see the Supplementary Methods.

### Reporting summary

Further information on research design is available in the Nature Research Reporting Summary linked to this article.

## Supplementary information

Supplementary Information

Description of Additional Supplementary Files

Supplementary Code 1

Reporting Summary

## Data Availability

These analyses re-used data provided by Keesey et al. *Nat. Commun*. 10, 1162 (2019)^4^, available through the Open Access Data Repository of the Max Planck Society, 10.17617/3.1D. Source data are provided with this paper.

## Source data

Source Data

## Code availability

All used code and a read-me file are available in the Supplementary Code 1 file.
