# Supplementary material for: Complexity of biological scaling suggests an absence of systematic trade-offs between sensory modalities in Drosophila
Source: Nat Commun. 2022 May 26;13:2944. doi: 10.1038/s41467-022-30579-y (PMC9135755; doi:10.1038/s41467-022-30579-y)
Supplement: Supplementary file 1 — Supplementary Information [file 41467_2022_30579_MOESM1_ESM.pdf]

Supplementary Information

Complexity of biological scaling suggests an absence of systematic trade-offs between sensory modalities in *Drosophila*

Authors: Max S. Farnworth and Stephen H. Montgomery

Table of contents

- Supplementary Methods
- Supplementary Note 1
- Supplementary Note 2
- Supplementary Note 3
- Figure S1
- Figure S2
- Figure S3

Supplementary Methods

To explore the scaling relationships of the head sensory structures (Figure 1/S1), , we conducted MCMC based (two-sided) phylogenetic regression models using BayesTraits V3.0.2[1]. We selected the 'Continuous: Regression' model and MCMC based calculation of the regression parameters with a Burn-in of 750,000 and 7.5 million iterations, sampling every 7500 iterations. For all models, we repeated the analysis twice to verify that the mean likelihood is very similar to the first model run. This was always the case, indicating that the MCMC analysis was stable. The species list was adapted so that species in the data were the same as species present in the tree. That specifically meant an exclusion of *D. mojavensis baja*, *D. sonorenisis* and *D. montium*, such that 59 species remained. We also decided to convert the original tree from a non-ultrametric tree to an ultrametric tree, as non-ultrametric trees have the tendency to cause biases of relatedness between longer and shorter branches. Importantly, though, the overall conclusions remained the same between the results using the original, non-ultrametric and our ultrametric tree. To convert the tree, the penalized likelihood approach was used as implemented in the "r8s" package[2,3], which included the number of total nucleotides that can change and three calibration time points (with time extracted from http://www.timetree.org/). "r8s" calculates the distance in time using the number of substitutions, the amount of character change from the original non-ultrametric tree and the time calibration points (as in [4]). For soe analyses, we pruned the phylogenetic tree with functions included in the *ape* and *geiger* package[5,6] (see Supplementary Code for details).

To test for isometric scaling between ESA (eye surface area) and FSA (funiculus surface area), we determined the posterior probability of the slope (β) by making use of MCMC calculations. MCMC makes iterative estimations of regression parameters including β, for which we calculated its mean, 95% confidence intervals, as well as the frequency of it being ≧1, i.e. its posterior probability, to test the hypothesis that $\beta = 1$ (Figure S1).

To explore scaling relationships of vision and olfaction in the brain and imaginal disc structures (Figure 2 and S2/S3), we used (two-sided) standard regressions in R (version 4.1.1[7]). To assess species dependent differences in scaling relationships in slope and intercept, we used *smatr*[8]. In single-species regressions, as well as pair-wise comparisons, we did not correct for multiple comparisons as there were several statistical limitations with the data, as noted in the main article, in particular a relatively low number of individuals per species. Plots were made using *ggplot2* included in the *tidyverse* package[9,10]. All code is available in the Supplementary Code.

To allow appropriate controlling for allometric scaling in the imaginal disc dataset we wanted to calculate a Rest of Disc value. For this, we determined a total disc size (Figure S3G) identical to how Keesey et al.[11] determined eye and antennal portion, i.e. by marking the outline

of a maximum projection of the disk image stack and determining the area size. By subtracting eye and antennal portion from the total size, we calculated the Rest of Disc (RoD) value.

Supplementary Note 1: Assessment of scaling relationships to qualify the use of ratios

Using ratios implies that the numerator (vision in Keesey et al.'s analyses[11]) scales in proportion to the denominator (olfaction), because it sets one size in proportion to the other. Proportional scaling assumes that the two traits scale isometrically[12], i.e., with a slope (β) of 1. If they do not scale with β=1, but instead scale hyper-allometrically (β>1), large eyed species (numerator) would appear to have enlarged eyes when measured with an EF ratio, as relative to the same eye size, olfactory size would be smaller if β>1. The reverse is true for hypo-allometric scaling (β<1) (see details in [12]). An alternative way to correct for allometric scaling artifacts is to include the two sensory domains in a function alongside an allometric control in a multiple regression (e.g., vision ~ body size + olfaction), as is common practice in allometric studies[13]. With such a setting the relationship of the two sizes in question could be assessed and the presence of an inverse relationship could be directly tested. Hence, our priority was to assess whether the different structures scaled isometrically. If not, using ratios would be inappropriate, in addition to the issue of limited interpretative power (Figure 1A).

In the full model *ESA ~ body length + FSA +* | *Phylogeny* (all variables $\log_{10}$-transformed) the posterior probability distribution showed that ESA does not scale isometrically with FSA (β=0.759, 95% confidence intervals (CIs) 0.009), but there is a significant relationship between ESA and FSA ($t_{df=54}$=6.124, P<0.001). However, body length did not scale with ESA ($t_{df=54}$=0.977, P=0.333) so was subsequently removed as an allometric control predictor, see section on alternative allometric controls below. In the subsequent regression model with identical settings, but without body length, β again indicated hypo-allometric scaling (Figure S1A; $\boldsymbol{\beta}$**=0.853**, 95% CIs 0.006; $t_{df=56}$=10.666, P<0.001), further illustrated by the β posterior probability distribution, where 94.33% of β iterations were lower than 1 (Figure S1A'). We therefore conclude that the use of ratios in this case is inappropriate, based on lack of isometry (Figure S1) and the potential to hide valuable information (Figure 1A).

Supplementary Note 2: Exploring clade specific effects

Using the phylogeny that was also displayed in the original Figure 1 in Keesey et al we subsetted the data to examine patterns within monophyletic clade. From the top of this figure to the bottom, we subsetted 4 groups: 1) *D.m. mojavensis* to *D. polychaeta* (24 species); 2) *D. cardini* to *D. immigrans* (9 species); 3) *D. sechellia* to *D. subobscura* (19 species); 4) *D. neocordata* to *D. sucinea* (6 species). The outgroup D. busckii was excluded. We performed the analogous analysis to the beginning of the head structure analyses, i.e. the model was ESA ~ BL + FSA + | Phylogeny. In all cases the results were similar to those described for the full dataset:

- Group 1: BL was an insignificant factor (β= 0.258, t_{19}= 0.553, P=0.586), and FSA scaled positively with ESA (β= 0.789, t_{19}= 4.032, P=0.001).
- Group 2: low power and large standard errors result in both predictors being insignificant (BL: β= -1.251, t_{4}= -0.829, P= 0.454; FSA: β= 0.904, t_{4}= 1.946, P= 0.123).
- Group 3: FSA was positively associated with ESA (β= 1.074, t_{14}= 3.464, P=0.004) and BL (β= 0.225, t_{14}= 0.645, P=0.529) was insignificant.
- Group 4: again, most likely due to low power with 6 species both predictors were insignificant (BL: β= 0.230, t_{1}= 0.127, P= 0.919; FSA: β= 0.754, t_{1}= 0.703, P= 0.610).

We also added group 1 and 2 to each other as these represent one group after the split between ancestral *Drosophila* and *Sophophora*, and added group 3 and 4. Here, FSA was positively associated with ESA (β= 0.788, t_{28}= 4.654, P< 0.001) and BL remained insignificant (β= -0.062, t_{28}= -0.138, P= 0.891). When combining group 3 and 4, FSA was positively associated with ESA (β= 1.033, t_{20}= 3.900, P= 0.001) and BL remained insignificant (β= 0.222, t_{20}= 0.703, P= 0.490). We could therefore not reveal any contradictory patterns to our original conclusions when considering smaller phylogenetic groupings.

Supplementary Note 3: Alternative allometric control – face width

We wanted to address the possibility that body length might be a suboptimal allometric control in our analyses as it did not scale significantly with ESA once FSA was included into the model (see beginning; *ESA ~ BL + FSA*). We used an alternative allometric control which was also previously employed, namely face width, or interocular space (IOS). We calculated this using the given values of head width, with eye width subtracted, and performed analogous models to the ones including BL described above. Using this alternative allometric control, we again did not find any support that would warrant the use of a ratio, nor did we find an inverse relationship. Specifically, scaling of ESA with IOS and FSA resulted into an insignificant, albeit nearly significant, relationship of IOS and ESA, and isometric relationship between ESA and FSA, but which then again would not be present upon exclusion of IOS (this would be identical to Figure S1A; ESA ~ IOS + FSA; β_{IOS}=-0.376, $t_{df=54}$=-1.987, P=0.052; β_{FSA}=0.987, $t_{df=54}$=9.413, P<0.001). Individual scaling of ESA with IOS and FSA with IOS resulted into a hypo-allometric and hyper-allometric relationship, respectively ($\beta_{ESA \sim IOS}$=0.813, $t_{df=56}$=3.683, P<0.001; $\beta_{FSA \sim IOS}$=1.191, $t_{df=56}$=6.736, P<0.001). Hence, the difference in slope estimates was even larger than when using body length (Figure 1B), and any such difference in allometric scaling again means that using a ratio is potentially problematic. A regression of the residuals of ESA and FSA revealed, similarly to body length, a positive significant relationship between residual ESA and FSA (β=0.979, $t_{df=56}$=9.407, P<0.001, as in Figure 1C).

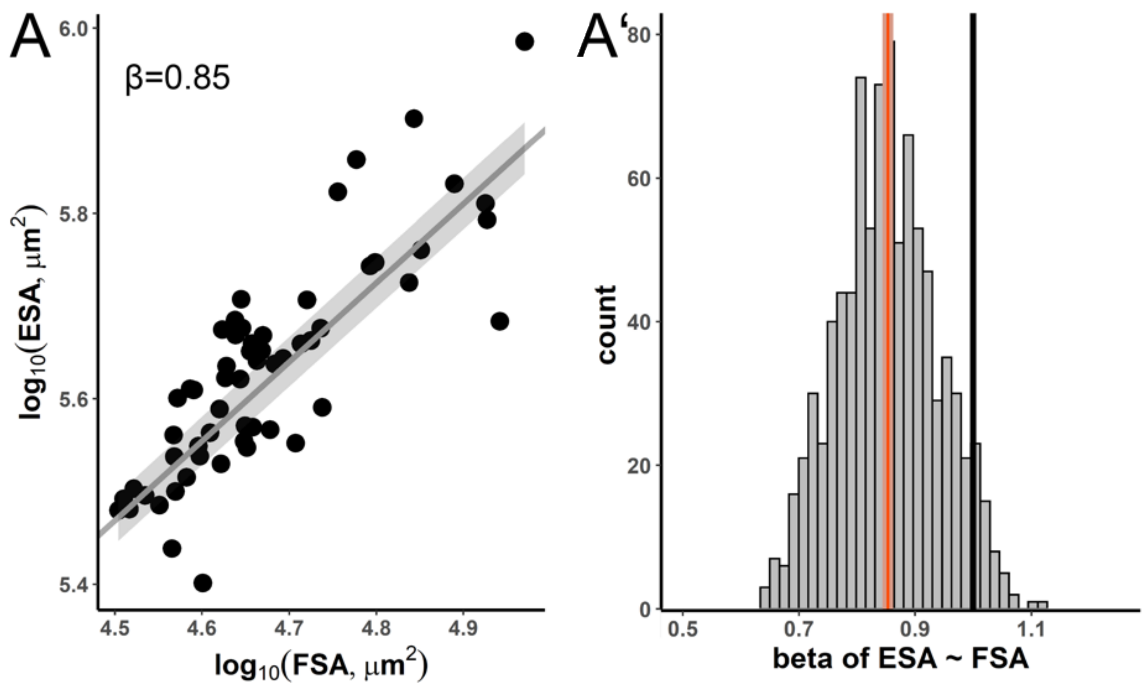

Figure S1: Analysing scaling relationships of eye and funiculus surface area (ESA and FSA) in 59 species reveals inadequacy to use ratios because of the lack of isometric scaling. **A**. ESA scales hypoallometrically (confidence intervals in grey bands) to FSA in a MCMC based phylogenetically controlled regression. **A'** A histogram of the slope from this MCMC analysis reveals the posterior probability of β, 94.33%. The mean is indicated in orange, with its confidence intervals. The black line indicates a value of 1, i.e. isometry. Source data are provided as a Source Data file.

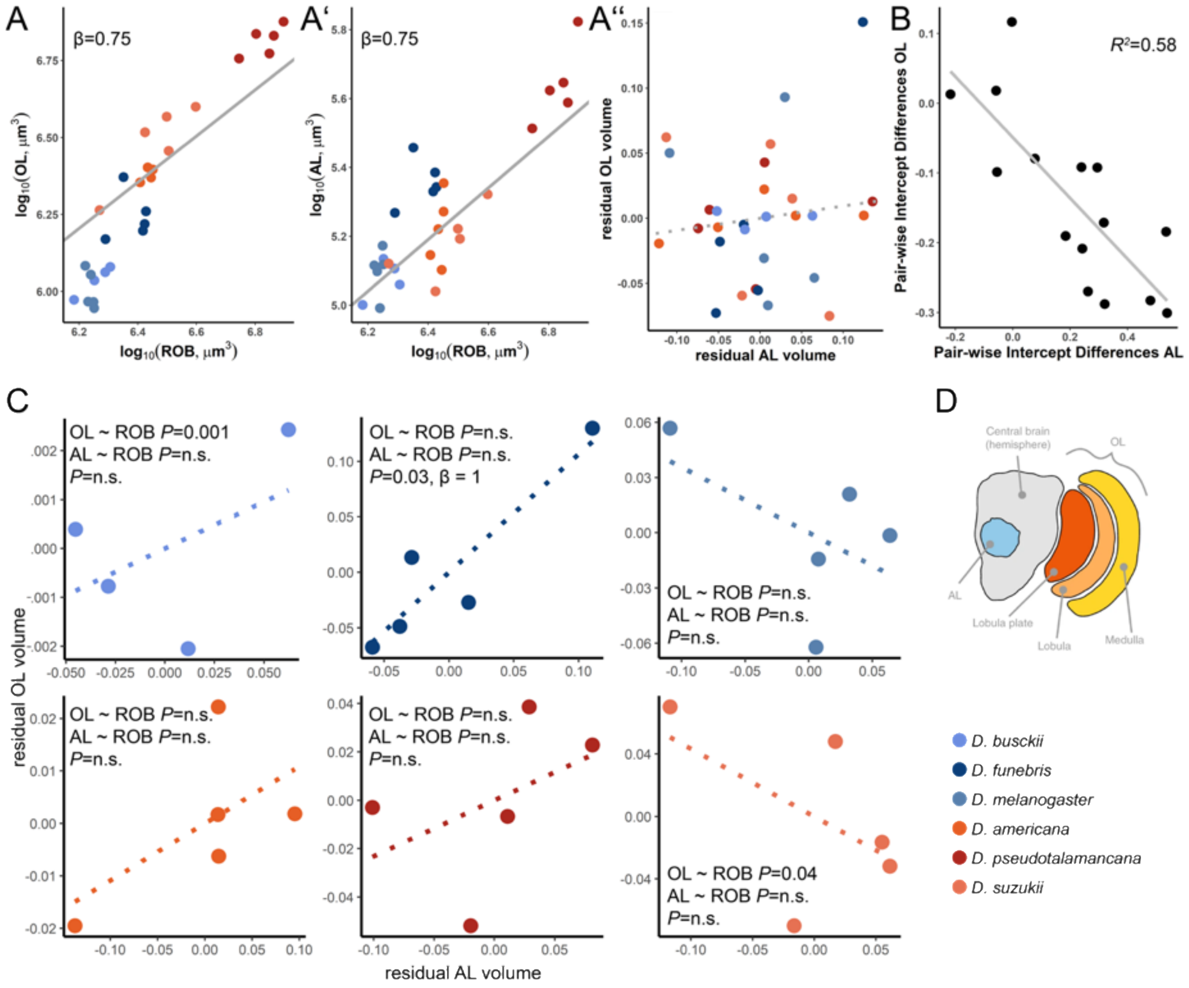

Figure S2: Additional plots for scaling relationships of brain neuropils (OL optic lobe, AL antennal lobe, ROB rest of brain). **A** and **A'** show the scaling relationships and slope of *OL ~ ROB + Species* and *AL ~ ROB + Species*, respectively. **A''** shows a plot of the residuals from A and A' with a non-significant, dashed regression line. **B** shows a plot of the intercept differences from the SMATR analysis for OL and AL in Figure 2 and a negative relationship between them. **C** shows species-specific regressions of the relative AL and OL sizes, that were generated from species-specific regressions. Indicated are whether these regressions as well as the regression of the residuals from the single regressions are significant based on the *F* statistic, and if so the slope value is indicated as well. **D** shows the volumes reconstructed by the original lines. Illustration is from the original publication by Keesey et al.[11]. Numbers of individuals per species are: *D. busckii*: *N*=4; *D. funebris*: *N*=5; *D. melanogaster*: *N*=5; *D. americana*: *N*=5; *D. pseudotalamancana*: *N*=5; *D. suzukii*: *N*=5. Source data are provided as a Source Data file.

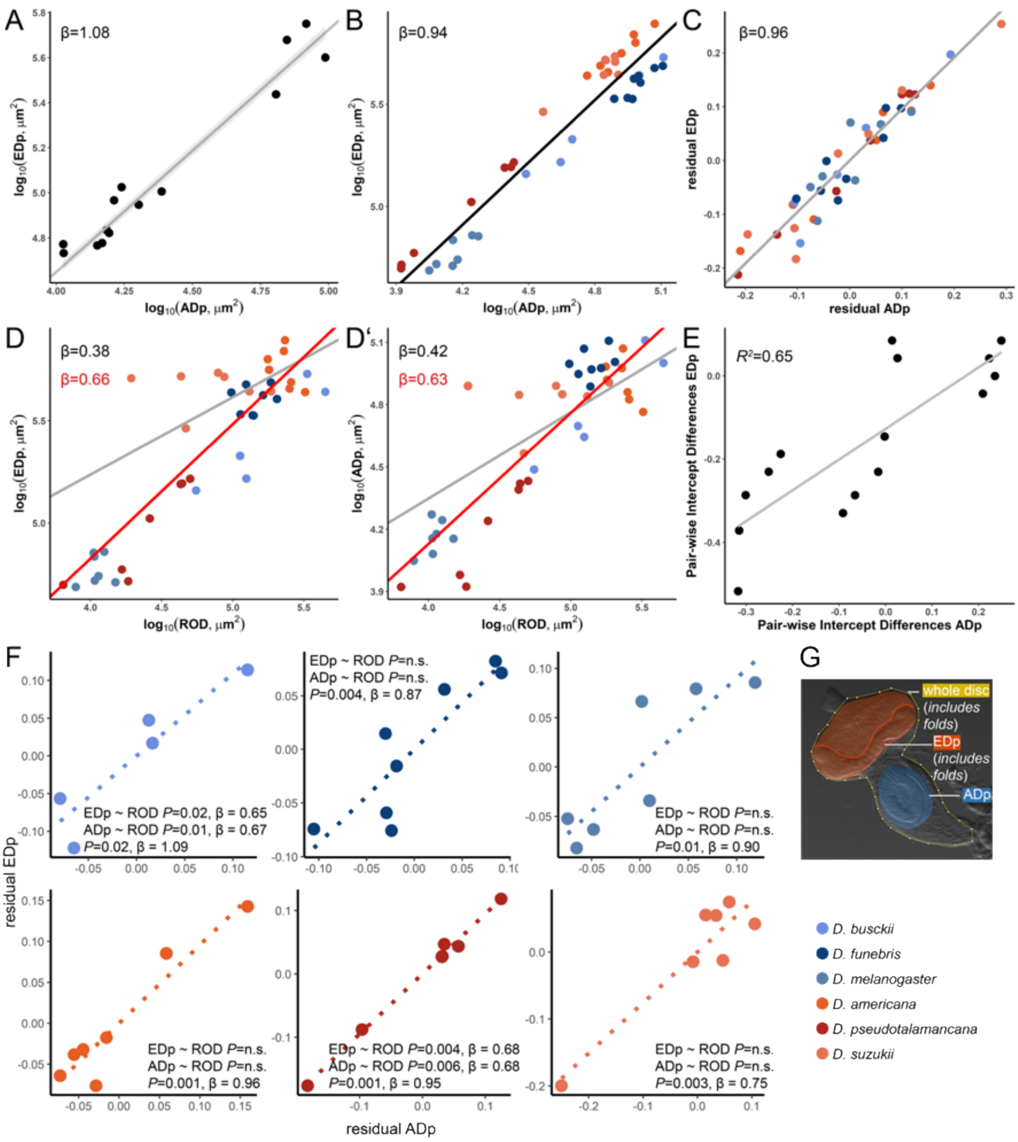

Figure S3: Additional plots for scaling relationships of imaginal disc portions (EDp=eye disc portion, ADp=antennal disc portion, ROD=rest of disc). **A** shows scaling of EDp and ADp in all of the 14 available species where assignment of individual data points to actual individuals was not necessary for species mean analysis. Shown is a MCMC based phylogenetically controlled regression**. B** shows the scaling relationships and slope of *EDp ~ ADp + Species* after having dropped the insignificant term ROD. **C** shows the scaling of the residuals of *EDp/ADp ~ ROD + Species* to display relative EDp and ADp size, confirming panel A and B. **D** and **D'** shows the scaling relationship of EDp and ADp, respectively, to ROD with species as factor. The red regression line is derived from a regression without species as factor. **E** shows a plot of the intercept differences from the smatr analysis for EDp and ADp in Figure 2 and a positive relationship between them. **F** shows species-specific regressions of the relative EDp and ADp sizes as well the regression significances based on the *F* statistic. **G** shows the different values, including a newly calculated rest of disc value. Figure of the original structure was taken from the Supplementary Information of the original publication by Keesey et al.[11]. Numbers of individuals per species are: *D. busckii*: *N*=5; *D. funebris*: *N*=8; *D. melanogaster*: *N*=7; *D. americana*: *N*=7; *D. pseudotalamancana*: *N*=7; *D. suzukii*: *N*=7. Source data are provided as a Source Data file.

Supplementary References

1. Meade, A. & Pagel, Mark. *BayesTraits* (2020). URL: http://www.evolution.rdg.ac.uk/BayesTraitsV3.0.2/BayesTraitsV3.0.2.html
2. Sanderson, M. J. Estimating absolute rates of molecular evolution and divergence times: a penalized likelihood approach. *Molecular Biology and Evolution* **19**, 101–109 (2002).
3. Sanderson, M. J. r8s: inferring absolute rates of molecular evolution and divergence times in the absence of a molecular clock. *Bioinformatics* **19**, 301–302 (2003).
4. Cicconardi, F. *et al.* Genomic signature of shifts in selection in a subalpine ant and its physiological adaptations. *Molecular Biology and Evolution* **37**, 2211–2227 (2020).
5. Paradis, E. & Schliep, K. ape 5.0: an environment for modern phylogenetics and evolutionary analyses in R. *Bioinformatics* **35**, 526–528 (2019).
6. Pennell, M. W. *et al.* geiger v2.0: an expanded suite of methods for fitting macroevolutionary models to phylogenetic trees. *Bioinformatics* **30**, 2216–2218 (2014).
7. R Core Team. R: A language and environment for statistical computing. *R Foundation for Statistical Computing, Vienna, Austria* (2021).
8. Warton, D. I., Duursma, R. A., Falster, D. S. & Taskinen, S. smatr 3– an R package for estimation and inference about allometric lines. *Methods in Ecology and Evolution* **3**, 257–259 (2012).
9. Wickham, H. *et al.* Welcome to the Tidyverse. *Journal of Open Source Software* **4**, 1686 (2019).
10. Wickham, H. *ggplot2: elegant graphics for data analysis*. (Springer New York, 2009).
11. Keesey, I. W. *et al.* Inverse resource allocation between vision and olfaction across the genus Drosophila. *Nat Commun* **10**, 1162 (2019).
12. Barton, R. A. & Montgomery, S. H. Proportional versus relative size as metrics in human brain evolution. *PNAS* **116**, 3–4 (2019).
13. Nunn, C. L. *The comparative approach in evolutionary anthropology and biology*. (The University of Chicago Press, 2011).
