# Supplementary material for: Complexity of biological scaling suggests an absence of systematic trade-offs between sensory modalities in Drosophila
Source: Nat Commun. 2022 May 26;13:2944. doi: 10.1038/s41467-022-30579-y (PMC9135755; doi:10.1038/s41467-022-30579-y)
Supplement: Supplementary file 2 — Description of Additional Supplementary Files [file 41467_2022_30579_MOESM2_ESM.pdf]

File Name: Supplementary Code 1
Description: READ ME CODE AND RESULTS of Farnworth & Montgomery "Complexity of biological scaling suggests an absence of systematic trade-offs between sensory modalities in Drosophila". The folder contains the following files here are sorted by dataset and figures. 1. "Script_Comment_MSF-SHM_vs2_publish": MAIN R-STUDIO SCRIPT CONTAINING ALL R-CODE. 2. "BayesTraits_exemplaryScript": EXEMPLARY SCRIPT TO BE ENTERED in Command line for BayesTraits. 3. Fig1_HeadStructures contains phylogenetic trees and related files from the phylogenetic analysis. 4. Fig2_Brain-Disc:code for all analyses of imaginal disc data. 5. "SupportingInfo": contains additional analyses that were (mostly) not displayed in figures but supplemented our conclusions, see Supplement for detail of results.
