# Supplementary material for: Complexity of biological scaling suggests an absence of systematic trade-offs between sensory modalities in Drosophila
Source: Nat Commun. 2022 May 26;13:2944. doi: 10.1038/s41467-022-30579-y (PMC9135755; doi:10.1038/s41467-022-30579-y)
Supplement: Supplementary file 3 — Supplementary Code 1 [file 41467_2022_30579_MOESM3_ESM.zip › Farnworth-Montgomery_STATS-CODE/SupportingInfo/Clade-Specific-Effects/Clade.docx]

**Clade-specific scaling effects in Drosophilidae**

To test the hypothesis that clade-specific differences in the scaling relationships exist that potentially could alter our conclusion of an absence of trade-offs we subsetted the head structure dataset into four groups. Using the phylogeny that was also displayed in the original Figure 1, our first group consisted of 24 species, from top to bottom, D.m. mojavensis to polychaeta. Group 2 consisted of 9 species from D. cardini to immigrans. Group 3 consisted of 19 species from D. sechellia to subobscura and Group 4 consisted of 6 species from D. neocordata to sucinea.

The outgroup D. busckii was excluded in this analysis.

We performed the analogous analysis to the beginning of the head structure analyses, i.e. the model was ESA ~ BL + FSA + | Phylogeny.

We found in group 1 very similar results with an overall likelihood of 34.306. BL was insignificant (*β*= 0.258, *t*_19_= 0.553, *P*=0.586), and FSA scaled positively with ESA (*β*= 0.789, *t*_19_= 4.032, *P*=0.001).

In Group 2 with 9 species, standard errors were quite large making both predictors insignificant (BL: *β*= -1.251, *t*_4_= -0.829, *P*= 0.454; FSA: *β*= 0.904, *t*_4_= 1.946, *P*= 0.123) The likelihood was low as well, with 7.739.

In Group 3 with a likelihood of 26.880, FSA was positively associated with ESA (*β*= 1.074, *t*_14_= 3.464, *P*=0.004) and BL (*β*= 0.225, *t*_14_= 0.645, *P*=0.529) insignificant.

In Group 4 with 6 species, standard errors were quite large making both predictors insignificant (BL: *β*= 0.230, *t*_1_= 0.127, *P*= 0.919; FSA: *β*= 0.754, *t*_1_= 0.703, *P*= 0.610). The likelihood was low as well, with 8.534.

Because of the low power for phylogenetic correction, we did two things. We added group 1 and 2 to each other as these represent one group after the split between ancestral Drosophila and Sophophora, and group 3 and 4. Group 1+2 had the likelihood of 40.958. FSA was positively associated with ESA (*β*= 0.788, *t*_28_= 4.654, *P*< 0.001) and BL remained insignificant (*β*= -0.062, *t*_28_= -0.138, *P*= 0.891). Group 3+4 had the likelihood of 36.904. FSA was positively associated with ESA (*β*= 1.033, *t*_20_= 3.900, *P*= 0.001) and BL remained insignificant (*β*= 0.222, *t*_20_= 0.703, *P*= 0.490).

So interestingly, there seem to be a differing scaling between ESA and FSA, hypoallometrically in group 1 and 2, but slightly hyperallometrically in group 3 and 4.

This shows that clade-specific analyses are important. However, we could not reveal any contradictory patterns to our original conclusions.

We also ran simple linear models with group 2 and 4 to see how the impression changed without phylogenetic correction, but no predictor were significant for ESA, and very similar values to the phylogenetic analysis.
