# Supplementary figures and images for: Complexity of biological scaling suggests an absence of systematic trade-offs between sensory modalities in Drosophila
Source: Nat Commun. 2022 May 26;13:2944. doi: 10.1038/s41467-022-30579-y (PMC9135755; doi:10.1038/s41467-022-30579-y)

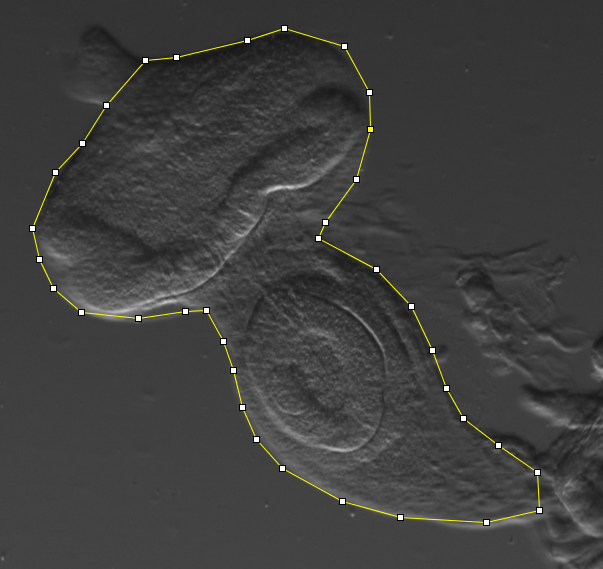

Supplement: Supplementary file 3 — Supplementary Code 1 [file 41467_2022_30579_MOESM3_ESM.zip › Farnworth-Montgomery_STATS-CODE/Fig2_Brain-Disc/Disc/wholedisc.png]
