# Supplementary material for: Complexity of biological scaling suggests an absence of systematic trade-offs between sensory modalities in Drosophila
Source: Nat Commun. 2022 May 26;13:2944. doi: 10.1038/s41467-022-30579-y (PMC9135755; doi:10.1038/s41467-022-30579-y)
Supplement: Supplementary file 4 — Reporting Summary [file 41467_2022_30579_MOESM4_ESM.pdf]

nature portfolio

Corresponding author(s): Max S Farnworth, Stephen H Montgomery

Last updated by author(s): Apr 26, 2022

Reporting Summary

Nature Portfolio wishes to improve the reproducibility of the work that we publish. This form provides structure for consistency and transparency in reporting. For further information on Nature Portfolio policies, see our Editorial Policies and the Editorial Policy Checklist.

Statistics

For all statistical analyses, confirm that the following items are present in the figure legend, table legend, main text, or Methods section.

n/a	Confirmed	
☐	☒	The exact sample size (n) for each experimental group/condition, given as a discrete number and unit of measurement
☐	☒	A statement on whether measurements were taken from distinct samples or whether the same sample was measured repeatedly
☐	☒	The statistical test(s) used AND whether they are one- or two-sided<br>*Only common tests should be described solely by name; describe more complex techniques in the Methods section.*
☐	☒	A description of all covariates tested
☐	☒	A description of any assumptions or corrections, such as tests of normality and adjustment for multiple comparisons
☐	☒	A full description of the statistical parameters including central tendency (e.g. means) or other basic estimates (e.g. regression coefficient) AND variation (e.g. standard deviation) or associated estimates of uncertainty (e.g. confidence intervals)
☐	☒	For null hypothesis testing, the test statistic (e.g. F, t, r) with confidence intervals, effect sizes, degrees of freedom and P value noted<br>*Give P values as exact values whenever suitable.*
☐	☒	For Bayesian analysis, information on the choice of priors and Markov chain Monte Carlo settings
☒	☐	For hierarchical and complex designs, identification of the appropriate level for tests and full reporting of outcomes
☒	☐	Estimates of effect sizes (e.g. Cohen's d, Pearson's r), indicating how they were calculated

Our web collection on statistics for biologists contains articles on many of the points above.

Software and code

Policy information about availability of computer code

Data collection	These analyses re-used data provided by Keesey et al. Nat. Commun. 10, 1162 (2019). We determined an additional value (total disc size) using their original data stacks. This is descriped in the Supplementary Methods. It uses standard tools in Fiji Version 2020-11-09 (https://imagej.net/software/fiji/). Besides this, no software was used for data collection.
Data analysis	All analyses were performed using open access statistical programs in R (Version 4.1.1; https://www.r-project.org) and BayesTraits V3.0.2 (http://www.evolution.rdg.ac.uk/BayesTraitsV3.0.2/BayesTraitsV3.0.2.html). In R we used additional packages: To assess species dependent differences in scaling relationships in slope and intercept, we used smatr (Warton et al. Methods Ecol Evol 3 (2012). Plots were made using ggplot2 included in the tidyverse package (Wickham et al. J. Open Source Softw. 4 (2019); Wickham Springer New York (2009).

For manuscripts utilizing custom algorithms or software that are central to the research but not yet described in published literature, software must be made available to editors and reviewers. We strongly encourage code deposition in a community repository (e.g. GitHub). See the Nature Portfolio guidelines for submitting code & software for further information.

Data

Policy information about availability of data

All manuscripts must include a data availability statement. This statement should provide the following information, where applicable:

- Accession codes, unique identifiers, or web links for publicly available datasets
- A description of any restrictions on data availability
- For clinical datasets or third party data, please ensure that the statement adheres to our policy

All data, code and analysis are available at following repositories.
Datasets of original publication: https://doi.org/10.17617/3.1D | Our code, results reports, etc are supplied in the Supplementary Code.

Field-specific reporting

Please select the one below that is the best fit for your research. If you are not sure, read the appropriate sections before making your selection.

- [x] Life sciences
- [ ] Behavioural & social sciences
- [ ] Ecological, evolutionary & environmental sciences

For a reference copy of the document with all sections, see nature.com/documents/nr-reporting-summary-flat.pdf

Life sciences study design

All studies must disclose on these points even when the disclosure is negative.

Sample size	As this is a matters arising piece the sample size was determined by the data collection by the original study's authors: Keesey et al. Nat. Commun. 10, 1162 (2019)
Data exclusions	we used all available data.
Replication	Experimental design was determined by the original study's authors
Randomization	Experimental design was determined by the original study's authors
Blinding	Experimental design was determined by the original study's authors

Reporting for specific materials, systems and methods

We require information from authors about some types of materials, experimental systems and methods used in many studies. Here, indicate whether each material, system or method listed is relevant to your study. If you are not sure if a list item applies to your research, read the appropriate section before selecting a response.

Materials & experimental systems

n/a	Involved in the study	
x		Antibodies
x		Eukaryotic cell lines
x		Palaeontology and archaeology
x		Animals and other organisms
x		Human research participants
x		Clinical data
x		Dual use research of concern

Methods

n/a	Involved in the study	
x		ChIP-seq
x		Flow cytometry
x		MRI-based neuroimaging
